# Research and application of a teaching platform for combined spinal-epidural anesthesia based on virtual reality and haptic feedback technology

**DOI:** 10.1186/s12909-023-04758-4

**Published:** 2023-10-25

**Authors:** Ting Zheng, Huihong Xie, Fei Gao, Cansheng Gong, Wei Lin, Peng Ye, Yuqing Liu, Bingwei He, Xiaochun Zheng

**Affiliations:** 1 Department of Anesthesiology, Shengli Clinical Medical College of Fujian Medical University, Fujian Provincial Hospital, Fuzhou, China; 2 Fujian Provincial Co-Constructed Laboratory of “Belt and Road”, Fujian Emergency Medical Center, Fuzhou, China; 3 Fujian Provincial Key Laboratory of Emergency Medicine, Fujian Emergency Medical Center, Fuzhou, China; 4 College of Mechanical Engineering, Fuzhou University, Fuzhou, China; 5 Department of Neurosurgery, Shengli Clinical Medical College of Fujian Medical University, Fujian Provincial Hospital, Fuzhou, China; 6 Fujian Provincial Key Laboratory of Critical Care Medicine, Fujian Emergency Medical Center, Fuzhou, China

**Keywords:** Combined spinal-epidural anesthesia, Virtual reality technology, Haptic feedback, Learning curve, Questionnaire

## Abstract

**Background:**

Intraspinal anesthesia poses significant teaching challenges and inadequate teaching resources, which ultimately limit students’ opportunities for practice. To address this issue, we aimed to develop a virtual platform for combined spinal-epidural anesthesia that merges virtual reality technology with haptic feedback technology, while assessing its educational impact and learning outcomes.

**Methods:**

We utilized MIMICS, 3Ds MAX, and UNITY 3D software to perform 3D reconstruction based on lumbar CT/MRI data from a standard male volunteer. The haptic coefficients were configured on each layer by 20 experienced anesthesiologists in accordance with the Geomagic Touch X force feedback device. A total of 20 anesthesiology interns completed 30 virtual puncture training sessions. Two experienced anesthetists evaluated the efficacy of the platform and the level of mastery achieved using the Global Rating Scale (GRS) and a Checklist score, respectively. Finally, a questionnaire survey was conducted to gather feedback on the virtual platform.

**Results:**

After the 10th session, the puncture time stabilized at 2.4 min. As the number of sessions increased, the Global Rating Scale (GRS) score stabilized by the 8th session, and the Checklist scores tended to stabilize by the 10th session. Results from questionnaires indicated that over half of the anesthesiology interns (70%) believed that the platform, which exhibited strong repeatability, improved their anatomical recognition and provided a strong sense of breakthrough in identifying the ligamentum flavum. The majority of them (80%) expressed satisfaction with the virtual platform.

**Conclusions:**

The platform effectively facilitated the acquisition of basic and accurate puncture skills on a virtual patient.

**Supplementary Information:**

The online version contains supplementary material available at 10.1186/s12909-023-04758-4.

## Background

Intraspinal anesthesia confers invaluable benefits over general anesthesia [1]. Therefore, it constitutes an indispensable aspect of the educational curriculum for anesthesiology interns. The successful performance of lumbar puncture mandates a confluence of anatomical knowledge and technical proficiency. Traditionally acquiring the technical expertise requires performing the procedure on a patient under close supervision initially, followed by a gradual transition to independence. Nonetheless, owing to the anesthesiology interns’ inexperience in the procedural domain, complications such as headaches and vascular injuries arising from incorrect puncture can be grave. Additionally, due to the procedure’s invasive and blind nature, opportunities for independent practice are infrequent.

Over the past decade, virtual reality (VR) environments have emerged as a promising solution for providing effective and safe training before conducting procedures on real patients, in a shorter period of time. VR has proven to be a valuable educational tool in various fields [2, 3]. To enhance the realism and applicability of virtual reality teaching systems for clinical procedures, reconstructing anatomical models and creating lifelike environmental scenes are essential. Furthermore, incorporating nuanced haptic feedback for different tissue types, such as skin, fat, and muscle, enables practitioners to gain a more authentic hands-on experience [4–6]. In the development of the virtual vertebral canal teaching model, Farber [7] proposed lumbar puncture in a VR environment using a haptic device with six degrees of freedom (6DOF). These systems featured a visual component that presented a 3D view of the entire lumbar spine, highlighting different needle positions during the puncture. The haptic component provided force feedback of the needle tip in various tissues during lumbar puncture. Lvquist [8] and Kulcsar [9] designed a virtual spinal anesthesia teaching model that offers 15 levels of adjustable force feedback, while Luca [10] developed a lumbar puncture simulator utilizing VR and a haptic device, providing more realistic haptic feedback during the needle insertion phase. However, none of these models simulate the complete human body and puncture scenario. Furthermore, the reconstructed procedural steps have certain limitations. To our knowledge, no reports have been published on a virtual system that combined spinal-epidural anesthesia simulation with comprehensive force feedback technology, encompassing the entire environment and procedure.

Therefore, we developed a virtual combined spinal-epidural anesthesia teaching platform by combining virtual reality (VR) and haptic feedback technology. The platform furnished an accurate 3D anatomical model of the lumbar spine and incorporated realistic haptic feedback corresponding to various tissue depths, facilitating the comprehensive simulation of the combined spinal-epidural anesthesia procedure within a secure operative setting. Subsequently, we conducted a pilot study and delivered virtual instruction to the anesthesiology interns, while assessing the effectiveness of the simulator and platform availability. Additionally, we assessed the impact of the virtual training on the students’ ability to master operational skills. This study provides useful guidelines for future training of students.

## Methods

### Graphics rendering

A 25-year-old male volunteer with a body mass index (BMI) of 20 kg·m^-2, no history of scoliosis surgery or trauma, and a normal lumbar spine by X-ray examination was included in the study. His lumbar computed tomography/magnetic resonance imaging (CT/MRI) dataset was used for three-dimensional (3D) reconstruction. The software used for image 3D reconstruction and processing was MIMICS 21.0. Threshold segmentation is capable of dividing an image into multiple regions based on the varied gray values of its pixels. The region growing technique commences from one or more seed points and expands the region iteratively by comparing the similarity of neighboring pixels with a predefined criterion until the stopping criterion is met. Image segmentation was performed using threshold segmentation and region growth method, and muscles, ligaments, spine, and nerves were extracted to create 3D models of each part. A smoothing tool was applied to adjust the models. After that, an STereoLithography (STL) file that describes the geometry of a 3D model, using a triangle mesh to represent the surface of an object, was exported for future use.

**Fig. 1:**
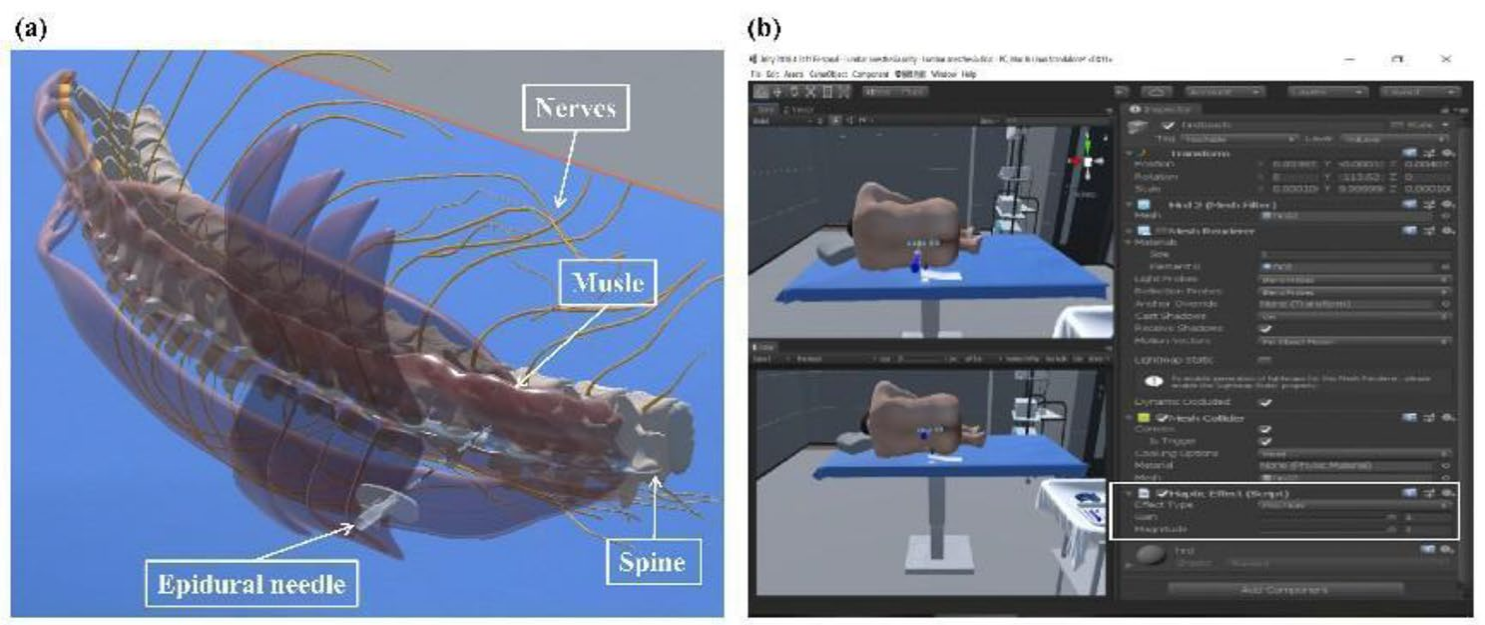
Obtained virtual scenes. (a)virtual lumbar puncture model;(b) force feedback coefficients scene

To integrate the 3D models of anatomical structures of lumbar levels 1–5, supraspinal ligament, interspinous ligament, ligamentum flavum, back muscle, and nerve, 3Ds MAX 2020 software was used. According to the anatomical atlas of the back and image data of healthy volunteers, the location distribution and thickness of each layer of the lumbar region were adjusted, and the skin map was drawn. Frame animations were created in 3Ds MAX, including draping, epidural catheterization, and catheter fixation, and the operating room environment was simulated. By hiding different hierarchies, the location of the puncture needle could be shown (Fig. 1a). The completed model was then converted into an FBX format file and imported into the UNITY 3D 2018 virtual reality development platform, where it was designed as an interactive virtual lumbar-epidural joint block model.

In addition, the model was designed to be viewed in stereoscopic mode to provide depth perception, which better emulated the operating room environment. To view the model in 3D, the trainee must use 3D glasses (HTC Vive, HTC Corporation, Taiwan), and the 3D monitor (Unity 3D, Unity Technologies, San Francisco, CA, USA) must support the side-by-side mode.

Figure 1. Obtained virtual scenes. (a)virtual lumbar puncture model;(b) force feedback coefficients scene.

### Haptics rendering

Twenty anesthesiologists, each having performed more than 200 spinal anesthesia procedures, participated in this study [11]. They were given 30 min to use a 3-degree-of-freedom haptic device, the Geomagic Touch X, to tune coefficients relative to tactile feedback. The haptic feedback was designed to provide real-time force feedback from the needle pathway, which helps in identifying various tissue layers encountered during the puncturing procedure. The needle movement was measured, and forces were applied as the needle was inserted through various tissue layers.

The sensory events included: (1) the force felt on the skin surface during disinfecting, (2) the force felt in the subcutaneous tissue as an epidural needle, (3) the force felt by the puncture needle on the supraspinal ligament, (4) the force felt by the puncture needle in the interspinous ligament, (5) the force of the puncture needle on the ligamentum flavum, (6) the force felt by the needle in the dura, and (7) the force of the needle touching the bone surface. We used the Friction Force Effects within the Unity 3D development software to simulate each sensory event during the development process. Specifically, we selected the friction effect type in Unity 3D (Fig. 1b). Friction Force Effects define a friction force applied to the haptic device in free space, determined by a gain (float from 0 to 1) and a magnitude (float from 0 to 1). Gain is the gain of the force; Magnitude represents the maximum force value and constrains the force from surpassing its prescribed limit as it increases. To achieve a more realistic force representation, we set the Magnitude to 1.

The Gain coefficients were adjusted in a white frame until the sensation was considered convincing within 0.1 unit (Fig. 1b). The force feedback’s Gain coefficients from 20 anesthesiologists for each sensory event were taken as the means (standard deviations) and converted into Newtonian force (N) after applying the force feedback device system prompt.

### Simulation training

This study involved twenty fifth-year anesthesiology interns, who were undergoing clinical internships at Fujian Medical University. The five-year undergraduate curriculum in anesthesiology at Fujian Medical University comprises four pre-clinical years focused on specialization theoretical knowledge (years 1 to 4), which also includes six months of clinical practice in year 4 at a clinical teaching unit in the hospital. The fifth year is dedicated to clinical clerkship to integrate theoretical and clinical competencies. After completing their undergraduate programs, all students were granted a Bachelor of Medicine and Bachelor of Surgery (MBBS) degree. Upon graduation, certain individuals decided to pursue a career as an anesthesiologist, while others chose to pursue advanced studies.

They all had no experience with vertebral canal puncture, some anesthesiology interns had learned about vertebral canal puncture or observed clinical operations, but none had experience in clinical or simulator puncture operations. After obtaining informed consent from each anesthesiology intern, they completed a form to assess their training experience in intraspinal anesthesia and analgesia. A 60-minute theoretical teaching session was provided to teach the median approach CSEA with PowerPoint (PPT) and video presentations. Each intern trained alone and avoided observing other interns during the experiment. After a teaching demonstration, the interns began to perform virtual puncture. Everyone trained five times a day for six consecutive days, with a short break of about 15 min each time, and completed 30 repetitions of puncture training.

The outcomes were evaluated by two experienced anesthetists using the Global Rating Scale (GRS) and Checklist scoring methodology [12]. The primary outcome was evaluated using the previously validated measurement tools: a 35-point GRS (Appendix 1) and a 10-item Checklist (Appendix 2). The total operating time from the determination of the puncture point to the fixation of the epidural catheter was also determined. The total operation time of 20 anesthesiology interns was used to fit the learning curve about the number of operations. The GRS score and Checklist score curves with the number of operations were made to evaluate the platform’s proficiency and interns’ learning effect.

After the training, each intern was asked to complete a specific questionnaire (Appendix 3) to rate their experience with the simulator, either single or multiple selected response items. A free-text box was also included to increase the richness of responses and allow for unexpected benefits or limitations of the models.

### Statistical analysis

Data were collected and analyzed using SPSS version 25 (SPSS Inc, IBM, Chicago, IL). Continuous variables, including force feedback’s Gain coefficients, total operation time, GRS score, and Checklist score, were expressed as mean ± standard deviation (*x̅* ± *s*), and data were rounded to one decimal place.

Interrater agreement was measured using the intraclass correlation coefficient (ICC), which quantifies the ratio of variance between participants due to error variance. The ICC values were interpreted as follows: 0.81 to 1.0, near-perfect agreement; 0.61 to 0.80, substantial agreement; 0.41 to 0.60, moderate agreement; 0.21 to 0.40, fair agreement; 0.00 to 0.20, slight agreement; and less than 0.00, poor agreement.

## Results

### Haptics coefficients setting

The force feedback coefficient was adjusted up and down by the 20 participating anesthesiologists, Gain coefficients data are represented as means (standard deviations). In the reconstructed back structure anatomy, the range of force is represented by the thickness of each anatomical feature, with varying feedback provided by different levels of structure to alter force dynamics along the needle pathway. The system then converted the Gain coefficients into Newtonian force, which corresponded to the thickness of each lumbar layer (Table 1).

**Table 1:** Thickness of each layer of lumbar, Force feedback’s Gain coefficients for 20 anesthesiologists and convert into Newtonian force. Gain coefficients data are represented as means ( standard deviations )

	skin	subcutis	supraspinal ligament	interspinal ligament	ligamentum flavum	epidural space	endorhachis	latissimus dorsi	Serratus posterior	longissimus and iliocostus	Multifi-dus	bone surface
Thickness; mm	2	8	5	35	5	8	2	7	5	7	6	10
Gain	0.3 (0.07)	0.4 (0.07)	0.5 (0.08)	0.4 (0.10)	0.6 (0.10)	0.1 (0.07)	0.2 (0.09)	0.3 (0.08)	0.3 (0.1)	0.4 (0.08)	0.3 (0.1)	1.0 (0.06)
Force feedback; N	2.1	2.8	3.5	2.8	4.2	0.7	1.4	2.1	2.1	2.8	2.1	7.0

**Fig. 2:**
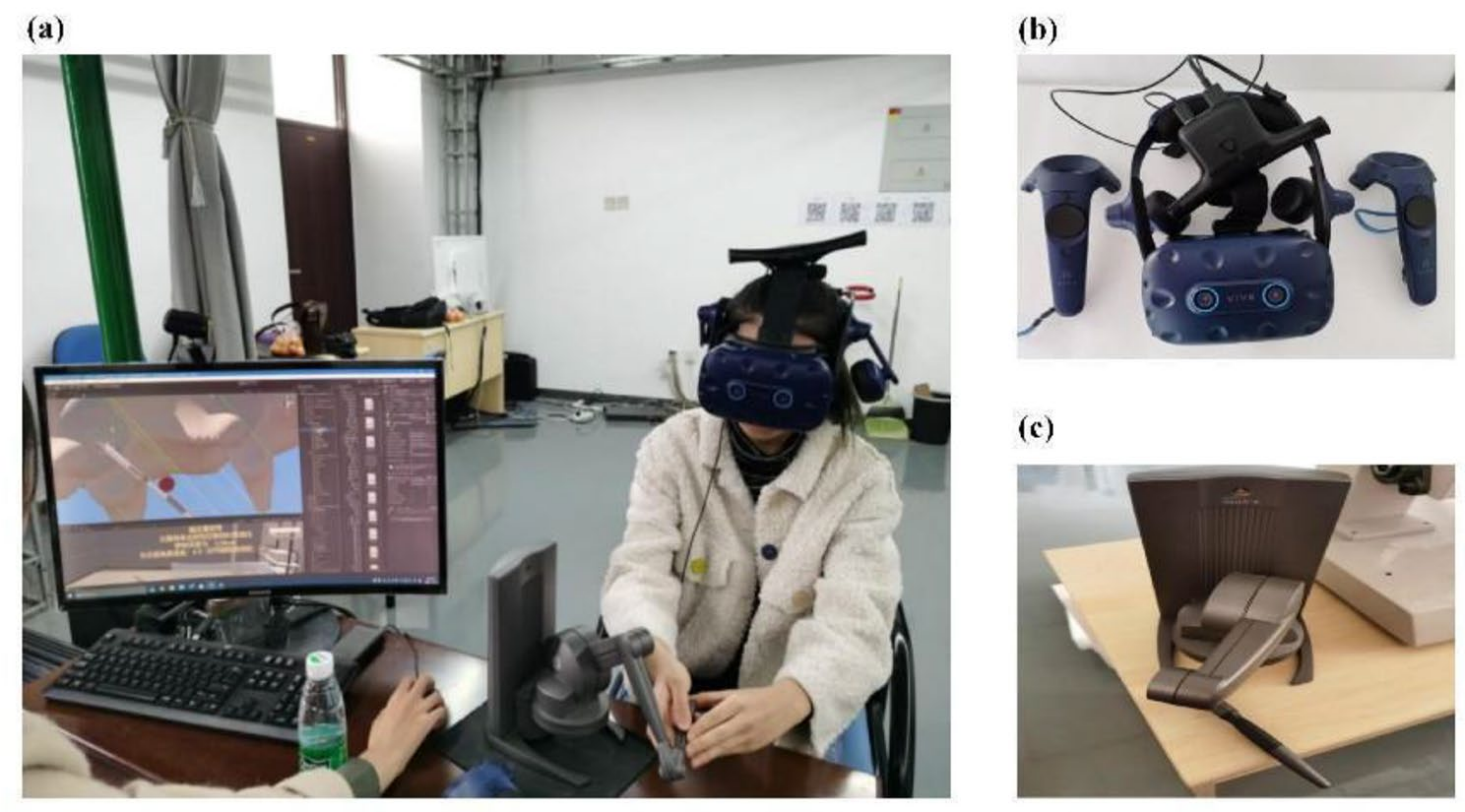
Virtual puncture operation method. **(a)** anesthesiology interns perform virtual punctures before a modified desktop platform;**(b)** VR glasses-HTC VIVE device; **(c)** Geomagic Touch X force feedback device

**Fig. 3:**
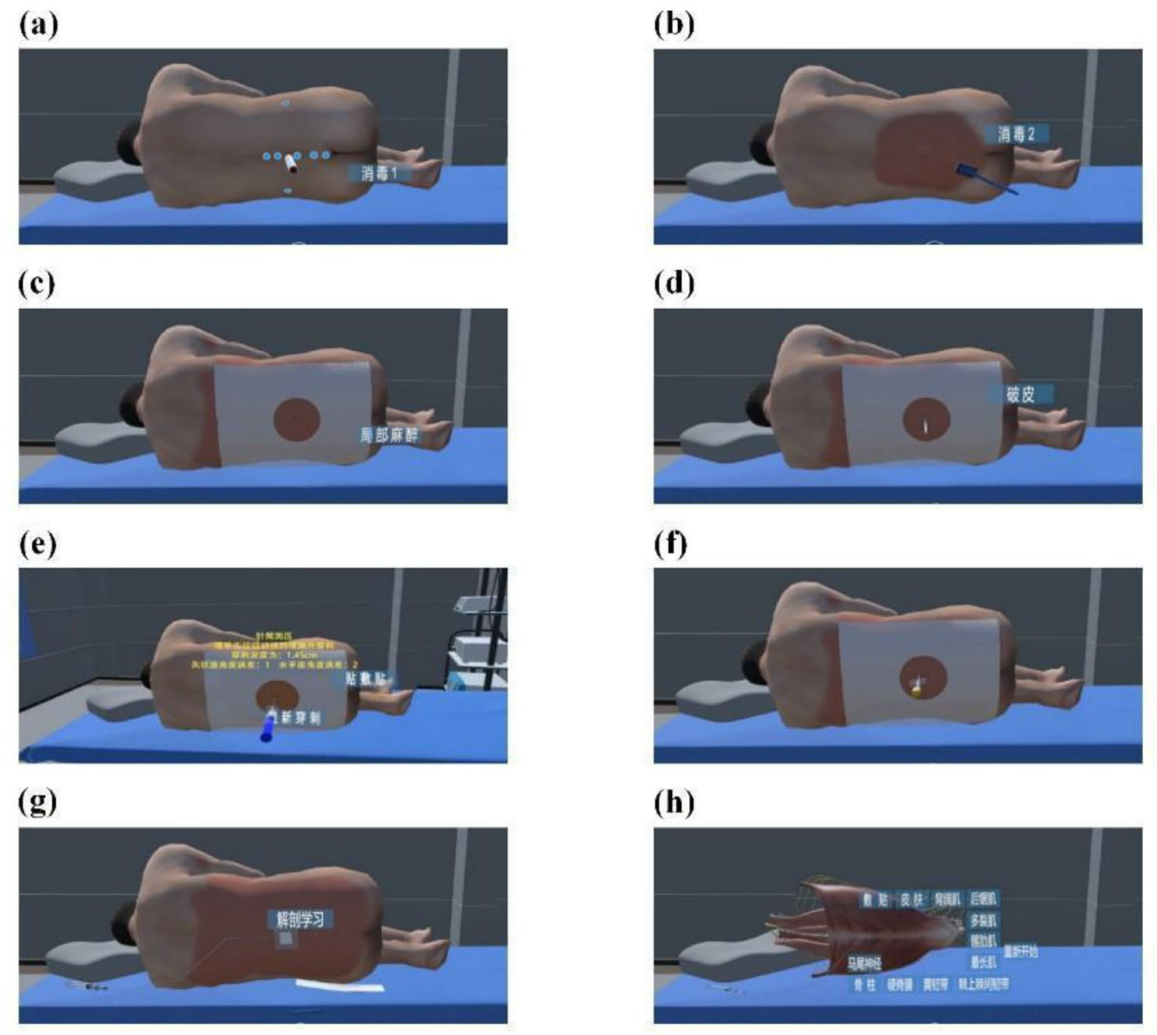
Virtual model operation process. **(a)**choose puncture point; **(b)** disinfect; **(c)** surgical drape; **(d)** skin dilatation; **(e)** epidural puncture; **(f)** spinal needle puncture; **(g)** epidural catheterization; **(h)** anatomical study

A virtual teaching platform for CSEA was established using virtual reality and haptic feedback technologies (Fig. 2a). Participants wore VR glasses (HTC VIVE device) (Fig. 2b) and sat in front of a modified desktop platform that displayed a 3D virtual model. A Geomagic Touch X force feedback device (Fig. 2c) simulated a median approach lumbar puncture on a virtual computer patient of standard weight and age. Each puncture step was designed according to clinical practice (Fig. 3a-g).

If the epidural needle touched the dura mater or bone surface, the system indicated a puncture error, and the operator was required to re-puncture. After the puncture, participants could learn about the anatomical position and shape of different back muscles by clicking the buttons of each anatomy level and strengthening their knowledge of related anatomy (Fig. 3h).

### Basic characteristics of anesthesiology interns

Basic characteristics, such as anesthesiology interns’ sex, age, and methods of learning about vertebral canal puncture, were recorded (Table 2). None of the 20 participants had previously performed vertebral canal punctures in clinical practice.

**Table 2:** Basic characteristics of 20 anesthesiology interns. Values are number (proportion) and mean (SD).

	Result n = 20
Sex;male	10(50%)
Age;y	22.5(0.9)
Rrevious learning about intraspinal puncture methods textbooks	16(80%)
human anatomy software	6(30%)
standardized puncture model	6(30%)
clinical observation	12(60%)
clinical practice	0(0%)

### Learning curve

**Fig. 4:**
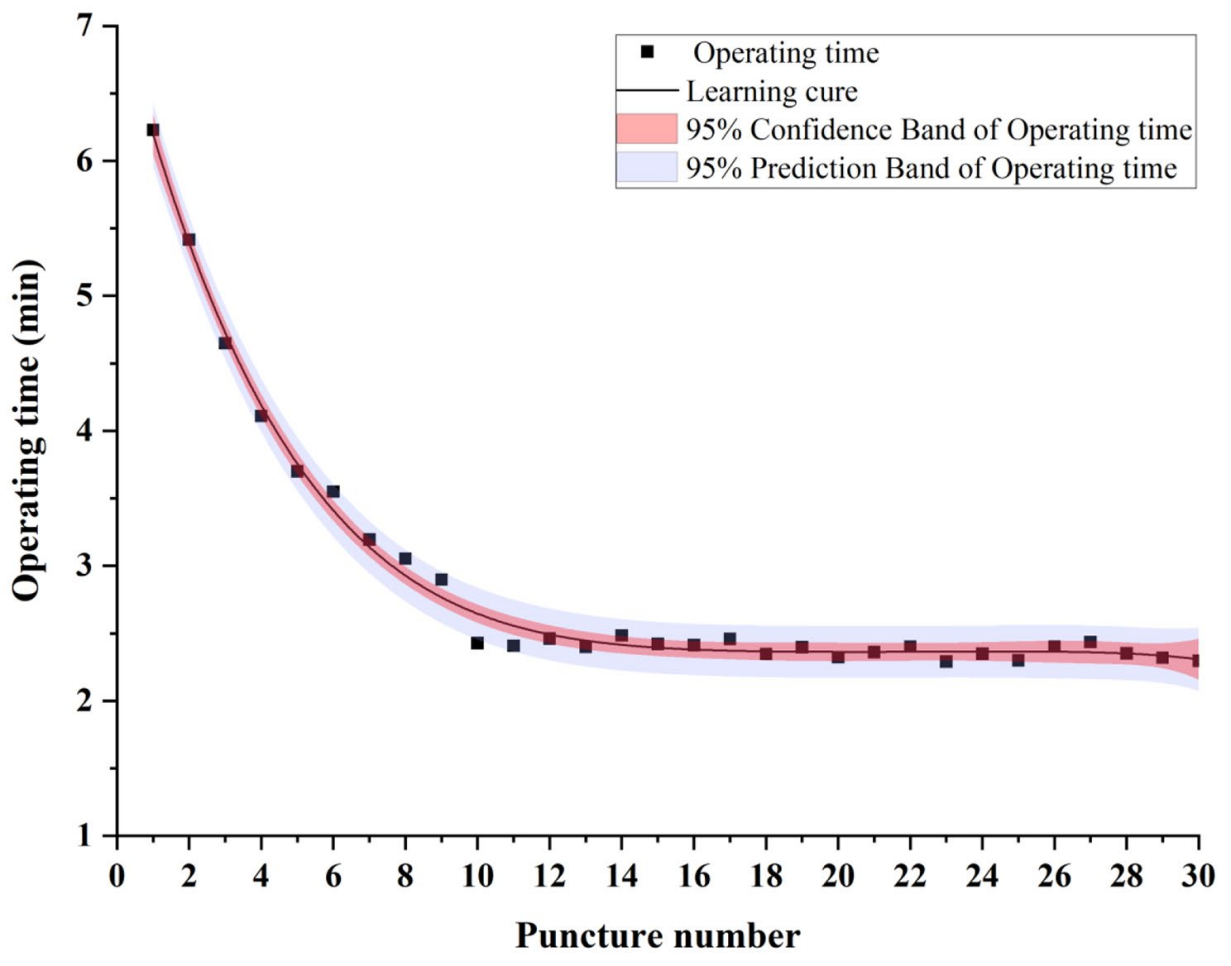
Learning curve of 20 anesthesiology interns over 30 puncture training attempts. Data are mean with 95% confidence band and prediction band

Among the 30 puncture operations per intern, the longest time was 10.2 min, and the shortest was 2.2 min. From the 10th time onwards, the operation time tended to stabilize, indicating that participants’ puncture skills reached proficiency at 2.4 min after ten training sessions (Fig. 4).

### GRS scores

**Fig. 5:**
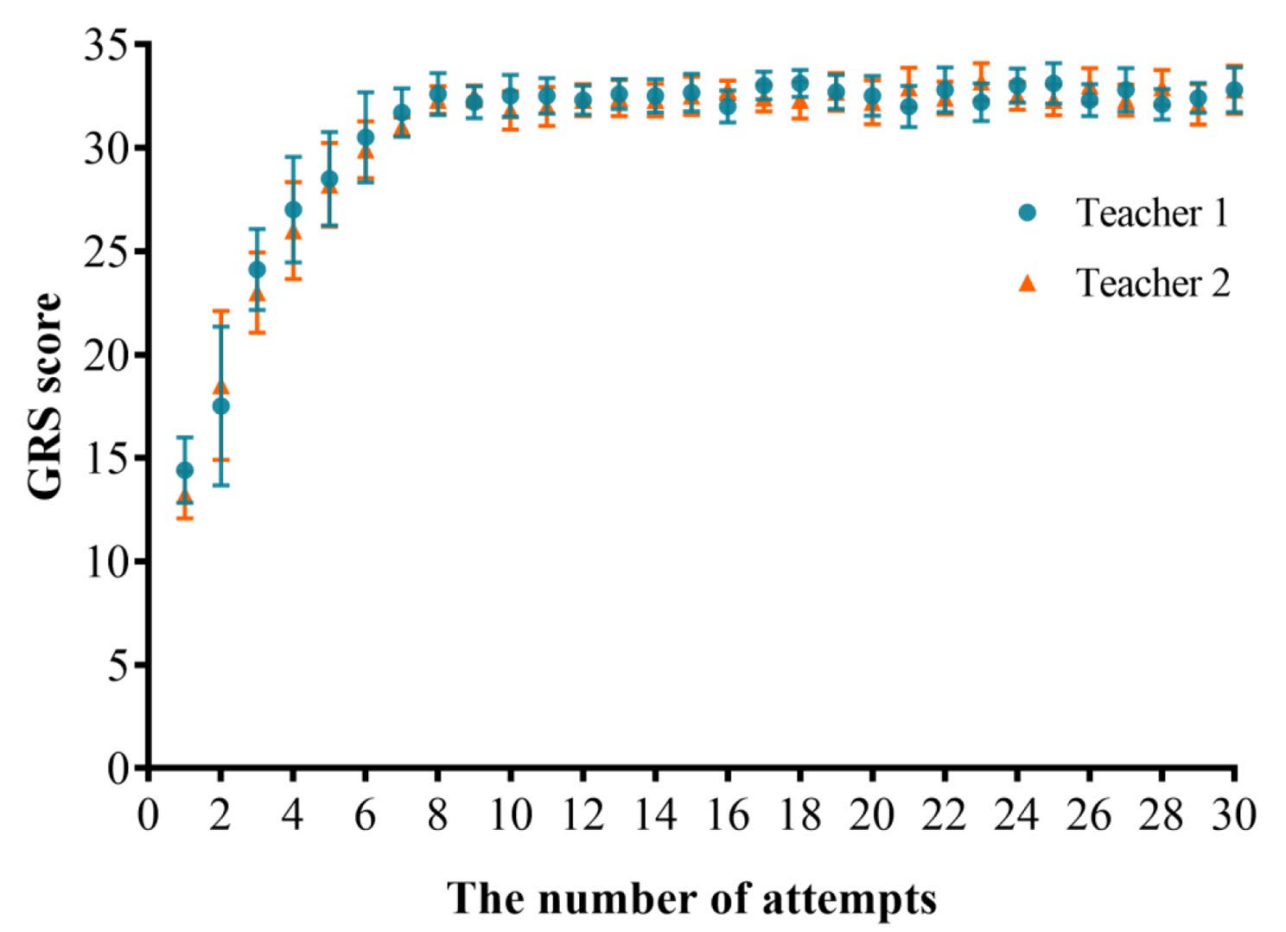
GRS score of 20 anesthesiology interns over 30 puncture training attempts. Data are mean with error bars showing SD. Teacher 1 ratings (blue circles); Teacher 2 ratings (orange triangles)

As the number of operations increased, the GRS score gradually increased and began to stabilize from the eighth time. After 30 operations, the average GRS score was 32.8 points (out of 35). The results showed that as the number of operations increased, interns’ fluency in the operation process improved, their movements became more proficient, and their understanding of relevant knowledge increased, indicating that our teaching platform was effective (Fig. 5).

For GRS scores, ICC and 95% confidence intervals were calculated for all 20 anesthesiology interns between the two experts, and the result was 0.970 (0.963–0.976) which indicated high consistency.

### Checklist scores

**Fig. 6:**
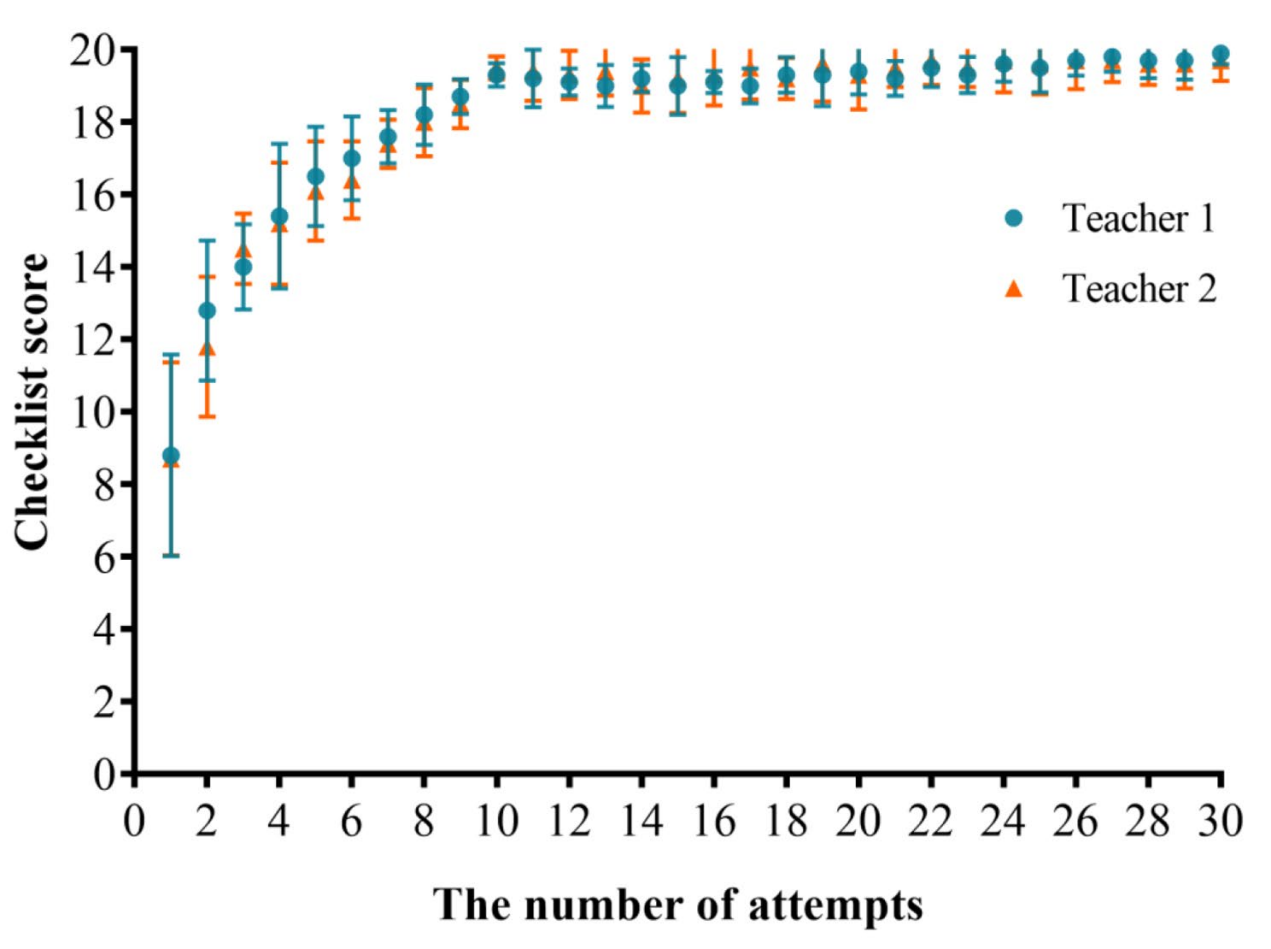
Checklist score of 20 anesthesiology interns over 30 puncture training attempts. Data are mean with error bars showing SD. Teacher 1 ratings (blue circles); Teacher 2 ratings (orange triangles)

As the number of operations increased, there was a gradual improvement in the Checklist scores. After the 10th operation, the operation scores became stable, and the average score of the Checklist score was 19.8 points (out of 20 points) after 30 operations. The results indicate that as the number of operations increased, the interns’ accuracy in completing the operation steps improved, and each operation step was complete and in place (Fig. 6).

For Checklist scores, ICC and 95% confidence intervals were calculated for all 20 anesthesiology interns between the two experts, and the result was 0.974 (0.967–0.981) which indicated high consistency.

### Questionnaire survey

**Fig. 7:**
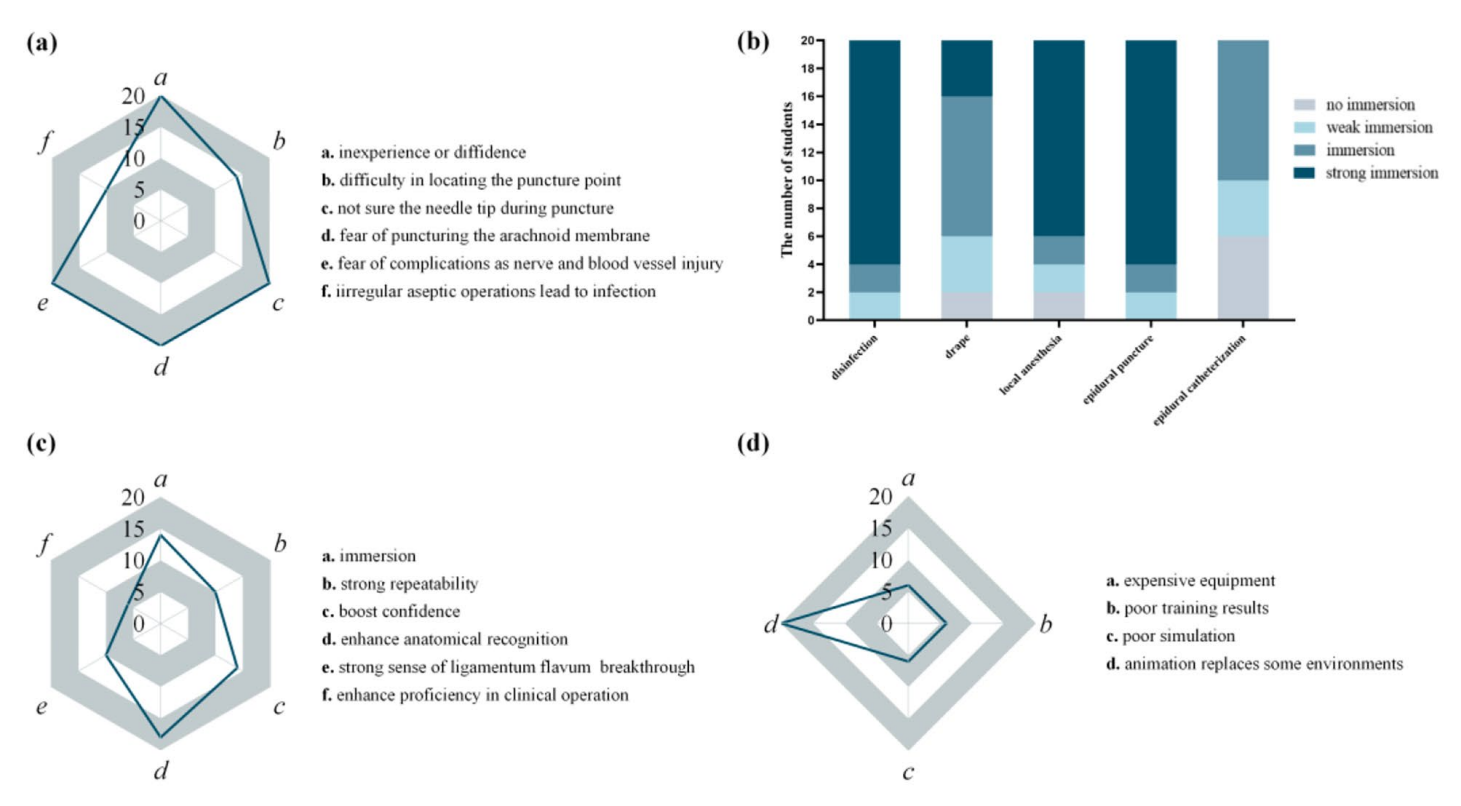
Questionnaire survey. **(a)** Number of anesthesiology interns indicating concerns about various aspects of clinical intraspinal puncture; **(b)** Anesthesiology interns’ clinical substitution in the virtual puncture; **(c)** Number of anesthesiology interns selecting various advantages of the virtual platform.; **(d)** Number of anesthesiology interns selecting various disadvantagesof the virtual platform

In this study, 20 anonymous questionnaires were distributed to the interns with a recovery rate of 100%. The anesthesiology interns were concerned about their lack of experience, confidence, and uncertainty about the needle tip during the puncture process. They feared puncturing the arachnoid membrane and the potential complications such as nerve and blood vessel injuries (Fig. 7a).

More than half of the anesthesiology interns (80%) using the virtual puncture platform thought that it was an excellent clinical substitute for disinfection and epidural puncture. Only two people did not feel the sense of clinical immersion during towel laying and local anesthesia (Fig. 7b).

More than half of the anesthesiology interns (70%) believed that the platform had solid repeatability, enhanced their anatomical recognition, and gave them a strong sense of breakthrough in the ligamentum flavum (Fig. 7c). Compared to performing on real patients in clinical practice, all the anesthesiology interns thought the drawback of platform was animation replaced some operation steps, thereby reducing the sense of engagement (Fig. 7d). In the satisfaction survey, two interns were very satisfied with the virtual intraluminal puncture platform, most of them (70%) were satisfied with it, and two interns were not satisfied.

## Discussion

In this study, we designed a new teaching platform that combined virtual reality with force feedback technology. The platform closely replicates the clinical operation by simulating the puncture procedure, offers a reasonable realism that helped the subjects in their training. This accurate representation enables effective training and skill development for medical professionals. The haptic epidural needle insertion simulator allows the safe practice of this technique in a risk-free environment for patients. The virtual simulator can serve as an educational precursor to the actual procedure on a patient. Our preliminary assessment demonstrated that trainees with prior experience found the simulator to be a realistic and valuable training tool that improved their confidence in performing the procedure.

Previous virtual reality models for vertebral canal puncture training, such as Farber’s [7], Lvquist’s [8], and Kulcsar’s [9] models, only provided students with a 3D view of the lumbar spine and a 2D view of human anatomy during the puncture process, without considering the patient’s whole body. In this study, we improved upon these models by using standard human data to reconstruct a 3D model of the lumbar spine. We then rendered the appearance of the entire human body, creating a standard left lateral lying mannequin designed explicitly for lumbar puncture training. By utilizing virtual reality and haptic feedback technology, we successfully simulated the entire operation process of CSEA. Furthermore, we incorporated the operating room environment to create a realistic scenario, enhancing immersion and bringing the simulation closer to a clinical setting. Upon completion, we asked 20 expert anesthesiologists to adjust the force coefficients for each layer, accurately replicating the tactile sensations experienced during an actual surgical procedure.

Munawar [4] proposed a fully immersive virtual reality system (FIVRS) for skull base surgery, which combines virtual reality (VR) with a 6DOF haptic device. This system provides force feedback that allows users to feel the differences between different tissue structures during the surgery. Carra [5] and Leung KM [6] also proposed haptic feedback models that focused on the force of a puncture needle on various tissue layers, such as skin, fat, muscle, and bone. These models provided force feedback at different tissue levels to help students identify the anatomical locations of the needle tip. Similarly, our study adopted a similar approach by providing different force feedbacks for different anatomical levels. It assisted students in recognizing the location of the needle tip, helped them adjust the puncture path.

Caitlin’s [13] study was conducted using hand motion analysis (HMA) to assess the acquisition and retention of technical proficiency among first-year medical students learning the lumbar puncture skill in a simulated setting. A total of 76% of subjects retained technical proficiency required a mean of 14 practices. In our study, the students’ operation time became stable after ten virtual punctures, which is reduced compared to Caitlin’s study. The main reason is, the virtual platform uses a standardized patient model for 3D reconstruction, and the focus of the puncture process is the anesthesiology interns’ experience of breaking through the ligamentum flavum. Repeated puncture without injury reduces the interns’ tension, enhances their confidence and understanding of virtual puncture, and helps them assist in achieving proficiency with minimal practice repetitions. However, compared to Naik [14] and Konrad’s [15] real patient studies, our study is necessary to investigate the extent to which improving virtual needle insertion skills can be translated into improved clinical operation skills in future studies.

Friedman [12] showed that the GRS score on overall operational fluency and the Checklist score on specific operational steps provided valid and reliable assessments of trainees’ ability to master skills, both in a simulated and clinical setting, distinguishing between different training stages of the process. Therefore, we use the above two scoring systems. GRS scoring mainly evaluates the accessibility of items. The Checklist score has been modified to subdivide the puncture process into ten specific steps, focusing on evaluating the accuracy of the steps. In addition, the intra-group correlation coefficient ICC for both the GRS and Checklist scores indicates a high consistency between the two experts.

Inexperienced practitioners often struggle to accurately adjust the needle tip angle at various levels during the completion of a ligamentum flavum breakthrough and cannulation. As a result, virtual puncture and force feedback simulation training on a platform can help to accumulate simulated clinical experience. Through this platform, interns can gradually eliminate skin, muscle, ligament, spine, and other structures layer by layer with the use of a handle. This method effectively corresponds with the VR interface and promotes anatomical understanding to improve unsuccessful operations. Interestingly, it can be obtained from the open text box in the questionnaire completed by the students that most students expressed a desire for this combined approach, including interactive videos and VR trainer, for educating trainees and trained personnel. This preference likely stems from the immersive, interactive, and innovative nature of multimedia learning, particularly with VR, which encourages active participation and engagement.

In conclusion, we have designed a virtual reality and haptic feedback technology simulator for conducting CSEA, which is a novel tool for enhancing procedural competency. Repetitive practice without causing injury can improve anesthesiology interns’ puncturing ability and potentially reduce patients’ discomfort and complications. Our data, albeit limited to a small number of trainees, supports the educational value of simulation training. We believe that virtual simulation has enormous potential to facilitate procedural training and will play an increasing role in anesthesiology.

### Limitation

However, our study has several limitations. First, the virtual 3D model is simulated based on a standardized patient, whereas clinical practice often involves more complicated cases such as scoliosis, ligament calcification, and spinal stenosis. Further improvements are necessary by incorporating these cases into the model for comprehensive training. Second, the authors find that the Gain coefficient of force feedback is inconsistent, resulting in a relatively small difference in parameters during operation, leading to no evident feeling of stiffness. Third, our study had a relatively small sample size, although statistical significance was achieved for the primary outcome variable. Fourth, certain steps, such as towel laying, inserting intraspinal needle, placing the epidural catheter, and affixing auxiliary materials, were replaced by animation during virtual training. This may lead anesthesiology interns to overlook these steps during actual operations. Additionally, due to the limitation of the force feedback device, aseptic operation has not been trained, leading to low awareness of aseptic operation. Fifth, the essential steps of communicating with patients and observing their reactions cannot be trained virtually.

### Conclusion and future directions

In summary, our independently developed virtual CSEA platform is a useful tool for guiding anesthesiology interns in puncture training. The platform effectively facilitated the acquisition of basic and accurate puncture skills on a virtual patient after several training sessions.

In our future research, we plan to further investigate a simulator with a virtual tactile glove, a device designed to simulate the sense of touch for a user wearing the glove. The glove may contain sensors, actuators or other devices that can produce tactile sensations, simulating the touch of a real object. Second, our simulator can accurately track the angle, depth, and duration of needle insertion at present. Future development will include an automatic data collection feature to facilitate personalized training programs. Third, our future research will involve enrolling students and dividing them into two groups, one receiving virtual model training and the other receiving clinical practice training, for a comparative analysis of their educational outcomes.

### Electronic supplementary material

Below is the link to the electronic supplementary material.

Supplementary Material 1

Supplementary Material 2

Supplementary Material 3

## Data Availability

The datasets analyzed during the current study are available from the corresponding author on reasonable request.

## List of abbreviations

GRS: Global Rating Scale
VR: virtual reality
6DOF: six degrees of freedom
BMI: body mass index
CT/MRI: computed tomography/magnetic resonance imaging
3D: three-dimensional
N: Newtonian force
ICC: intraclass correlation coefficient
CSEA: combined spinal-epidural anesthesia
