# Supplementary material for: Research and application of a teaching platform for combined spinal-epidural anesthesia based on virtual reality and haptic feedback technology
Source: BMC Med Educ. 2023 Oct 25;23:794. doi: 10.1186/s12909-023-04758-4 (PMC10601272; doi:10.1186/s12909-023-04758-4)
Supplement: Supplementary file 1 — Supplementary Material 1 [file 12909_2023_4758_MOESM1_ESM.docx]

**Appendix 1**

Global Rating Scale(GRS)^[8]^ :(35-point)

| **Preparation for Procedure** | **1**  Did not organize equipment well. Has to stop procedure frequently to prepare equipment. | **2** | **3**  Equipment generally organized. Occasionally has to stop and prepare items. | **4** | **5**  All equipment neatly organized, prepared, and  ready for use |
| --- | --- | --- | --- | --- | --- |
| **Respect for tissues** | **1**  Unnecessary force on tissue/caused damage | **2** | **3**  Careful tissue handling; occasional inadvertent damage | **4** | **5**  Consistent appropriate tissue handling; minimal damage |
| **Time& Motion** | **1**  Unnecessary moves | **2** | **3**  Efficient time/motion; some unnecessary moves | **4** | **5**  Clear economy of movement & max. Efficiency |
| **Instrument Handling** | **1**  Repeated tentative/awkward moves with instruments | **2** | **3**  Competent instrument use; occasionally stiff/awkward | **4** | **5**  Fluid moves with instrument; no awkwardness |
| **Flow of Procedure** | **1**  Frequent procedure stops; unsure of next move | **2** | **3**  Demonstrated some forward planning with reasonable progression | **4** | **5**  Obvious plan of procedure with effortless flow of one move to next |
| **Knowledge of Procedure** | **1**  Deficient knowledge | **2** | **3**  Knew all important steps | **4** | **5**  Demonstrated familiarity with all aspects |
| **Overall Performance** | **1**  Very poor | **2** | **3**  Competent | **4** | **5**  Clearly superior |
