# Supplementary material for: Research and application of a teaching platform for combined spinal-epidural anesthesia based on virtual reality and haptic feedback technology
Source: BMC Med Educ. 2023 Oct 25;23:794. doi: 10.1186/s12909-023-04758-4 (PMC10601272; doi:10.1186/s12909-023-04758-4)
Supplement: Supplementary file 2 — Supplementary Material 2 [file 12909_2023_4758_MOESM2_ESM.docx]

**Appendix 2**

| **Item** | **0**  **correct** | **1**  **partial correct** | **2**  **quite correct** |
| --- | --- | --- | --- |
| 1.Confirm the puncture site. Correct alignment |  |  |  |
| 2.Disinfect for 3 times and place drape (scope, sequence) |  |  |  |
| 3.Infiltrates subcutaneous layers with local anesthetic |  |  |  |
| 4.Inserts needle layerby layer,advance needle slowly while applying pressure on the plunger |  |  |  |
| 5.Touch ligamenta flavum |  |  |  |
| 6.Feels for a “pop” sensation as it passes ligamenta flavum |  |  |  |
| 7.Not through the dura mater |  |  |  |
| 8.Puncture with a fine needle and pull out the core |  |  |  |
| 9.Detaches the syringe and threads the catheter to a depth of 4-5 cm |  |  |  |
| 10.Secure the catheter and apply the dressing |  |  |  |

Checklist^[8]^ :(20-point)
