# Supplementary material for: Research and application of a teaching platform for combined spinal-epidural anesthesia based on virtual reality and haptic feedback technology
Source: BMC Med Educ. 2023 Oct 25;23:794. doi: 10.1186/s12909-023-04758-4 (PMC10601272; doi:10.1186/s12909-023-04758-4)
Supplement: Supplementary file 3 — Supplementary Material 3 [file 12909_2023_4758_MOESM3_ESM.docx]

**Appendix 3**

Virtual combined spinal-epidural anesthesia teaching platform questionnaire

Dear students: Hello, in order to explore the training of this virtual intraspinal puncture teaching platform, we would like to collect your comments and suggestions on this platform. This survey will be conducted in a confidential manner, which will not affect you personally. Your information and answers will be kept confidential. Please do not have any concerns. Please tick “√” in front of the best answer. Thank you for your support and help.

1. Your identity

◯Intern

◯First year resident

◯Second year resident

◯Third year resident

2.How did you learn the knowledge about intraspinal puncture before this virtual training? (Multiple choice)

□Book

□Anatomy software

□Standardized puncture model

□Clinical observation

□Clinical operation

□Virtual intraspinal puncture teaching platform

3.If you have learned the knowledge of intraspinal puncture through clinical operation, how many patients have you operated?

◯never

◯1-2 cases

◯3-4 cases

◯5-6 cases

◯7-8 cases

◯9 cases

4.If you were to perform intraspinal puncture on patient, what would you worry about? (multiple choice)

□No experience and lack of confidence

□Difficulty in locate the puncture site

□The position of the needle tip during blind puncture was not sure

□Worried about puncturing the arachnoid membrane

□Complications as nerve and blood vessel injury

□Aseptic operation is not standard, resulting in infection

1. How do you feel about the clinical substitution of disinfection during the operation of the virtual teaching platform?

◯no immersion

◯weak immersion

◯immersion

◯strong immersion

1. How do you feel about the clinical substitution of draping during the operation of the virtual teaching platform?

◯no immersion

◯weak immersion

◯immersion

◯strong immersion

1. How do you feel about the clinical substitution of local anesthesia during the operation of the virtual teaching platform?

◯no immersion

◯weak immersion

◯immersion

◯strong immersion

1. How do you feel about the clinical substitution of epidural puncture during the operation of the virtual teaching platform?

◯no immersion

◯weak immersion

◯immersion

◯strong immersion

9. How do you feel about the clinical substitution of epidural catheterizaion during the operation of the virtual teaching platform?

◯no immersion

◯weak immersion

◯immersion

◯strong immersion

10.What advantages do you think virtual intraspinal puncture has compared with traditional teaching models (textbook, observation operation, standardized model, clinical operation)? (multiple choice)

□Immersive

□Strong repeatability

□Enhance piercing confidence

□Enhanced anatomical recognition

□Enhance the sensation of breaking through the ligamenta flava

□Enhance the fluency of the whole operation process

11.What do you think about the disadvantages of the virtual platform? (multiple choice)

□Expensive equipment

□Difficult to familiarize with operating equipment

□Cumbersome

□Poor training effect

□Poor simulation effect

□Force feedback distortion (too weak or too strong)

□Animation replaces some environments

1. What do you think of the biggest difficulty in the virtual process? (multiple choice)

□Localization difficulty

□Difficulty in adjusting the angle of the puncture needle

□Difficult to break through the ligamentum flavum

□The operation steps did not connect smoothly

13.Are you satisfied with the virtual puncture platform?

◯very satisfied

◯satisfied

◯general

◯dissatisfied

◯very dissatisfied

14.Do you have any other comments on this virtual teaching platform?
